# “Secular trends (2001–2020) in physical fitness as a health component in physiotherapy students from Bydgoszcz (Poland)”

**DOI:** 10.1038/s41598-024-62157-1

**Published:** 2024-05-20

**Authors:** Andrzej Lewandowski, Marcin Siedlaczek, Zuzanna Piekorz, Łukasz Kryst

**Affiliations:** 1 Department of Physiotherapy, Faculty of Health Sciences, Ludwik Rydygier Collegium Medicum in Bydgoszcz Nicolaus Copernicus University, Bydgoszcz, Poland; 2 Department of Anthropology, University of Physical Education in Kraków, Kraków, Poland; 3 Department of Physiotherapy, Faculty of Health Sciences, Ludwik Rydygier Collegium Medicum in Bydgoszcz, Nicolaus Copernicus University, Toruń, ul. Świetojańska 20, 85-094 Bydgoszcz, Poland

**Keywords:** Male female, Physical fitness, Skinfold, Secular trend, Environmental dependencies, Anatomy, Health care, Health occupations

## Abstract

The aim of this study was to assess changes in the components of physical fitness that are conducive to the health of young people surveyed between 2001 and 2020. Hypotheses were formulated about an increase in the thickness of skinfolds, especially among women, the deterioration of the results of fitness tests and the lack of conditioning of the examined changes with socioeconomic factors. Every year, physiotherapy students at the Collegium Medicum in Bydgoszcz, Nicolaus Copernicus University in Toruń (Poland) were examined for body height and mass, skinfold thickness, flexibility, static strength, abdominal muscle strength, functional strength and endurance. The results were obtained from 1161 female students and 464 male students. Similar trends were observed for most of the studied characteristics in people of both sexes. In male students, secular trends towards a decrease in the thickness of biceps skinfolds (R^2^ = 0.455, p = 0.002) and lower leg skinfolds (R^2^ = 0.314, p = 0.015) were found. In female students, secular trends towards an increase in the body mass (R^2^ = 0.41, p = 0.003), a decrease in the thickness of skinfolds over the biceps (R^2^ = 0.477, p = 0.001) and decreased flexibility (R^2^ = 0.283, p = 0.023) were found. Male sex and the thickness of suprailiac skinfolds were frequent predictors of the, assessed motor abilities and socioeconomic factors did not significantly affect their maintenance. The obtained results, except for a few secular trend examples of the somatic features (male student’s age and calf skinfold, female student’s body mass, male and female student’s biceps skinfold) and flexibility in women, showed their stability and the lack of conditioning by social factors and by the fitness test. Attention to the appropriate level of the observed components is justified, especially in light of the identified trend that showed their deterioration.

## Introduction

Health is a concept that has many aspects, and its understanding often depends on the professional or scientific specialization of the person defining it^1,2^. Regardless, it is widely expected to exist and, at the same time, is carelessly neglected by a large part of society, despite abundant information about the significant contributions of health determinants that are dependent on us^2^. It is also known that physical fitness supports health, especially by maintaining an appropriate level of flexibility, static and functional strength and endurance, as well as indicators of targeted physical activity^3^. In some professions, the motor aspect of physical fitness is necessary for their implementation. For people engaged in medical professions, fitness components, especially health-promoting ones, should be a model to be followed by a large part of society. However, it is known from numerous studies that the physical fitness of current generations is deteriorating or changing its structure and that motor skills are transferred to systems other than the musculoskeletal system^4–8^. An extensive analysis of scientific literature on secular trends of long-term somatic and motor development revealed that these changes differ in terms of the direction and intensity and largely depend on the study period, sex and age of the participants, as well as on demographic conditions^9–12^. Scientific reports about secular trends in somatic features and the results of student motor tests confirm their different intensities and even directions in terms of individual characteristics while revealing other causes of their occurrence^9,13–15^. Research on the motor development of physiotherapy students shows that their fitness differs from that of students at physical education universities and other universities involved in education in this field. Some of its components deteriorate^16–19^. However, most of scientific reports on changes in physical fitness result from cross-sectional and comparative studies and less often from long-term studies of secular trends. Therefore, undertaking additional studies seems to be justified, especially in terms of the heterotelic approach to the interpretation of the obtained results^20^.

### Purpose of the research

In connection with the above, we attempted to assess changes in the components of physical fitness of young people studying physiotherapy, which with a high probability can be a reference for the population of young, healthy and fit people. This statement is based on the results of the research on the assessment of physical fitness of young women studying physiotherapy and students of other faculties of a medical university, as well as on our own observations of public opinion on the work and profession of a physiotherapist^21,22^. Further goals included the assessment of secular trends in physical fitness that support health among students, as well as to determine predictors for these trends.

### Hypothesis

Based on numerous reports about the deteriorating health of young generations, especially in relation to obesity, and negative changes in physical fitness, also found in the components supporting motor achievements of the currently studied group^19^, a hypothesis was put forwards about the occurrence of secular trends towards an increase in the thickness of skinfolds, especially among women, the deterioration of the results of fitness tests and the lack of conditioning of the examined changes with socioeconomic factors. It was also assumed that the directions of secular trends of the examined characteristics in male students would be similar to those among female students. Their verification and the statements resulting from the previous study will allow for the fullest possible assessment of the secular trends in physical fitness of the observed physiotherapy students in accordance with the concept presented by the authors of Eurofit^23^.

## Materials and methods

During the years 2001–2020, cross-sectional studies were carried out on physiotherapy students from the Collegium Medicum in Bydgoszcz, which has been part of the Nicolaus Copernicus University in Toruń since 2004. The Ludwika Rydygier Medical Academy, which was the original name of the university, was the first of the Polish medical academies to offer education in the field of physiotherapy. Bydgoszcz is a large provincial city located in the west-central part of Poland, with more than 350,000 population and with academic traditions. The research was carried out during classes; it was initially conducted in the third year of long-term master’s studies and later in the first and second years of bachelor’s studies. The change in the study model did not allow the research to be carried out in 2014, and beginning in 2019, the research included male and female master’s students. Therefore, the location of the subject “movement education and movement teaching methodology” in the study program determined the stage of the study at which the research was carried out and, thus, also determined the age of the students. Until 2010, during recruitment to studies, a fitness test was carried out, which included measuring the static strength, endurance and agility, as well as assessing swimming skills. With the exception of the last group of male and female students, whose research period coincided with the pandemic and lockdown, all surveyed students took part in physical education classes (60 h), movement education with movement teaching methodology (110 h) and summer and winter camps (110 h).

### Test procedures

At the end of each year of the observation, the body height and mass were measured. These results and socioeconomic characteristics, as well as the basic information about the study process presented above, were used in the article on the components of physical fitness conducive to achieving motor achievements^19^. The division of the material into two studies was dictated by indications for a new, instrumental (heterotelic) interpretation of the results of physical fitness testing proposed by a large group of EUROFIT authors, as well as Polish authorities on human motor skills research^20,23^. The decision to present the material in two separate works was also influenced by its considerable volume, which, if contained in one article, could make it illegible or require selective presentation. Hence the similarity in the description of the basic somatic features (height and body weight) and socioeconomic conditions of the studied groups. The majority of the surveyed male and female students were residents of large cities (65.97%), a large proportion of whom were from Bydgoszcz (20.25%), and almost all of them were graduates of general secondary schools (95.75%). The parents of some surveyed students received secondary education (fathers, 35.82%; mothers, 42.03%), and a large percentage, especially among mothers, completed higher education (42.58%).

Basic somatic characteristics were supplemented with thickness measurement of triceps, biceps, subscapular, abdominal, suprailiac and calf skinfolds^24^. Measurements of the body height and the thickness of the skinfolds were made using an anthropometer, with an accuracy of 1 mm, and a calliper, with an accuracy of 0.2 mm, from the Swiss company GPM. The body mass was determined using an electronic scale manufactured by DB-1H Castex in China, with an accuracy of 0.01 kg. The Eurofit test was used to examine the health-promoting components of physical fitness in the following areas: flexibility (sit and reach [SAR]), static strength of the right and left hands (handgrip [HGR]), trunk strength (sit-ups [SUP]) and cardiorespiratory endurance (endurance shuttle run [ESR]). Considering the structure of motor skills, which are a component of physical fitness or are used to describe it, the tests conducted reflected the level of fitness (energy) abilities and only indirectly reflected information (coordination) abilities^5,20,25^. The Eurofit test is a randomized, widely used tool based on simple motor tests that do not require special motor skills. The commencement of the motor tests was preceded by the information about the method and purpose of the test and by a short warm-up. To measure the static force, an SH 5001 hydraulic hand dynamometer from Saehan Corp., Masan, Korea, which enables measurements with an accuracy of 1 kG, was used. With the exception of the last group, in which students, owing to the pandemic and lockdown, carried out the measurements and motor tests themselves, all previous tests were performed by a specialist research team under the same conditions and in accordance with the test procedures. The results were recorded in accordance with testing recommendations^23^.

Inclusion in the study group was based on the membership in the observed student group, a good general condition, and personal informed consent of the participants. The most common reason for the exclusion from the study was absence from classes or an injury that prevented performance of motor tests. In total, sets of results were obtained from 464 male and 1161 female students, constituting nearly 95% of the students involved in the research. The characteristics covered by the research were considered by taking into account basic socioeconomic characteristics, such as the size of the place of residence, the type of school completed and parents’ education, as well as the inclusion of a fitness exam in the recruitment procedure for studies.

### Ethics

The research was carried out with the consent of the Bioethics Committee at the Collegium Medicum im. Ludwika Rydygiera of Nicolaus Copernicus University in Bydgoszcz (No. KB 44/2004-extended every 4 years until 2020). All men and women were informed about the purpose of the study, the type and duration of the effort, and the possibility of withdrawing from the study without giving any reason. After this information was provided, the students provided informed consent to participate in the study. All procedures contributing to this work followed the ethical standards of the relevant national and institutional human experimentation committees and the 1975 Declaration of Helsinki, as amended in 2008.

### Statistics

The results of the measurements are presented in tables containing average values and standard deviations. The relationships of the changes in the average annual values of the examined characteristics between women and men were assessed with Pearson’s linear correlation coefficients. The values of the coefficients were interpreted as the strength of the linear relationship between the characteristics. Formally, it is the normalized covariance of two defined variables. Changes in the measured values over time were assessed using simple linear regression analysis to determine the regression coefficients so that the model could predict the value of the dependent variable. Analysis of the impact of features on fitness results was carried out using the multiple linear regression method, which is used to fit linear or linearized models between one dependent variable and more than one independent variable.

The following characteristics were used as potential predictors: body height and mass, thickness of skinfolds, fitness exam in the recruitment procedure for studies, sex, age, place of residence, type of completed school, and father’s and mother’s level of education.

Statistical analysis was performed at the Statistical Analysis Center of Nicolaus Copernicus University in Toruń using the Python programming language (3.8.10) with the following libraries: statsmodels (0.13.1), pandas (1.3.4), scikit-learn (1.1.1), matplotlib (3.5.0), and seaborn (0.11.2).

## Results

Table 1 summarizes the sizes of the studied groups of students and the values of basic somatic features^19^. The descriptive statistics indicate that in all years of observation, women accounted for significantly larger shares of the sample, with slightly lower average ages and standard deviations. The men surveyed in 2020 were characterized by a greater average body mass than that in earlier years.

**Table 1:** Demographic characteristics of the participants.

Study year	n (%)			Age (mean ± SD)		Body height (cm)		Body mass (kg)	
	All	Men	Women	Men	Women	Men	Women	Men	Women
2001	47	12 (26)	35 (74)	22.08 ± 2.27	21.37 ± 0.73	180.70 ± 5.20	165.74 ± 5.15	77.33 ± 10.57	59.11 ± 7.49
2002	40	13 (32)	27 (68)	21.15 ± 0.69	21.11 ± 0.51	183.51 ± 5.54	169.25 ± 6.65	75.62 ± 5.39	60.78 ± 7.37
2003	38	6 (16)	32 (84)	23.33 ± 1.37	22.69 ± 1.26	182.33 ± 5.30	165.89 ± 7.37	87.17 ± 10.61	59.81 ± 9.78
2004	47	8 (17)	39 (83)	23.00 ± 2.14	22.18 ± 0.72	180.09 ± 3.89	166.07 ± 6.34	80.88 ± 9.42	58.56 ± 9.01
2005	58	11 (19)	47 (81)	22.09 ± 0.30	22.11 ± 0.52	175.25 ± 6.40	164.15 ± 5.90	68.64 ± 9.83	59.91 ± 11.44
2006	60	8 (13)	52 (87)	23.12 ± 1.55	22.12 ± 0.43	177.38 ± 11.84	165.44 ± 5.07	77.25 ± 13.88	59.60 ± 6.79
2007	116	37 (32)	79 (68)	21.54 ± 1.02	21.70 ± 0.85	179.60 ± 5.69	167.82 ± 6.32	75.44 ± 10.05	61.59 ± 9.47
2008	61	21 (34)	40 (66)	22.14 ± 2.83	21.30 ± 0.72	180.31 ± 7.18	166.69 ± 5.94	79.00 ± 9.81	58.55 ± 6.73
2009	176	69 (39)	107 (61)	21.03 ± 0.94	20.96 ± 0.88	180.99 ± 6.08	166.72 ± 6.23	77.10 ± 10.62	60.36 ± 8.95
2010	112	33 (30)	79 (70)	20.79 ± 2.51	20.24 ± 1.19	179.75 ± 6.82	167.61 ± 6.10	76.33 ± 9.52	58.49 ± 7.46
2011	80	28 (35)	52 (65)	20.61 ± 1.20	20.23 ± 0.51	177.89 ± 6.84	166.62 ± 4.98	76.64 ± 8.44	60.37 ± 10.05
2012	96	30 (31)	66 (69)	20.43 ± 0.90	20.29 ± 0.76	180.45 ± 5.92	166.50 ± 5.87	79.05 ± 15.97	60.49 ± 8.13
2013	98	31 (32)	67 (68)	20.71 ± 1.35	20.25 ± 0.77	181.45 ± 6.74	167.97 ± 5.75	76.76 ± 11.47	60.87 ± 9.23
2015	113	30 (27)	83 (73)	21.23 ± 0.57	21.33 ± 0.77	179.07 ± 6.63	168.01 ± 6.16	78.67 ± 11.50	61.11 ± 8.00
2016	114	26 (23)	88 (77)	21.27 ± 0.60	21.25 ± 0.65	180.85 ± 7.94	165.86 ± 5.52	82.44 ± 15.66	61.14 ± 9.78
2017	101	26 (26)	75 (74)	21.19 ± 0.40	21.19 ± 0.54	178.79 ± 5.66	166.18 ± 5.81	81.88 ± 10.87	63.99 ± 11.94
2018	99	23 (23)	76 (77)	21.30 ± 0.93	21.22 ± 0.67	179.71 ± 6.70	167.16 ± 5.75	79.72 ± 10.48	61.66 ± 12.03
2019	83	26 (31)	57 (69)	21.54 ± 1.03	21.30 ± 0.76	178.63 ± 4.56	166.91 ± 5.75	79.74 ± 12.12	61.92 ± 8.65
2020	86	26 (30)	60 (70)	21.77 ± 1.07	21.15 ± 0.61	183.23 ± 5.38	168.15 ± 5.76	84.93 ± 12.86	61.09 ± 10.07

n (%), statistical characteristic and percentage values of the size of the surveyed student groups; mean, average age of the student groups.

*SD* standard deviation.

### Results of measuring somatic features

Table 2 presents the results of the skinfold thickness measurements for the groups of male students, and Table 3 presents the results for the groups of female students.

**Table 2:** Skinfold thickness of the examined men (mean ± SD).

Study year	Triceps skinfold	Biceps skinfold	Subscapular skinfold	Abdominal skinfold	Suprailiac skinfold	Calf skinfold
2001	15.83 ± 4.41	11.00 ± 3.52	17.08 ± 6.16	14.17 ± 6.31	26.33 ± 12.14	17.75 ± 5.43
2002	11.08 ± 3.04	6.69 ± 2.72	13.15 ± 3.72	13.00 ± 6.70	20.15 ± 6.08	12.92 ± 3.07
2003	8.17 ± 2.48	16.00 ± 5.80	13.00 ± 4.56	22.50 ± 14.90	16.50 ± 14.61	14.67 ± 5.47
2004	6.75 ± 3.92	14.25 ± 7.17	12.50 ± 5.58	25.50 ± 18.31	14.12 ± 10.92	15.38 ± 9.01
2005	5.09 ± 1.45	12.09 ± 3.99	11.27 ± 4.54	34.45 ± 13.62	9.18 ± 3.82	15.09 ± 5.07
2006	5.12 ± 2.42	14.88 ± 8.39	8.88 ± 3.14	27.88 ± 10.48	8.75 ± 4.62	11.38 ± 4.63
2007	5.32 ± 1.94	11.86 ± 4.12	10.49 ± 4.76	14.46 ± 7.64	8.51 ± 4.81	11.70 ± 4.07
2008	5.19 ± 1.89	12.86 ± 4.29	10.29 ± 4.04	12.14 ± 4.97	8.10 ± 3.99	13.43 ± 4.47
2009	4.86 ± 1.35	11.43 ± 5.12	9.77 ± 3.99	9.91 ± 4.46	7.94 ± 4.31	10.46 ± 3.29
2010	5.09 ± 1.16	11.76 ± 4.98	10.52 ± 3.87	10.30 ± 5.43	12.09 ± 8.00	11.18 ± 3.16
2011	6.07 ± 3.25	13.61 ± 5.49	11.11 ± 4.88	12.11 ± 5.86	12.18 ± 6.30	13.43 ± 5.17
2012	10.43 ± 4.17	6.87 ± 3.22	12.87 ± 4.78	12.33 ± 5.86	11.87 ± 4.95	13.13 ± 4.45
2013	11.19 ± 4.07	6.68 ± 2.99	13.13 ± 4.57	15.35 ± 7.13	12.74 ± 4.85	13.45 ± 3.21
2015	11.10 ± 4.99	6.40 ± 3.69	13.07 ± 6.76	15.90 ± 11.18	13.97 ± 9.40	13.40 ± 5.16
2016	12.88 ± 4.85	6.38 ± 2.56	15.77 ± 10.27	21.08 ± 13.79	18.62 ± 10.70	15.00 ± 4.67
2017	12.54 ± 4.54	7.00 ± 2.90	16.73 ± 6.76	23.46 ± 11.84	20.38 ± 9.58	12.35 ± 4.37
2018	9.70 ± 4.42	6.30 ± 2.14	14.70 ± 6.61	18.17 ± 8.28	17.04 ± 9.58	9.22 ± 3.12
2019	10.08 ± 5.25	6.35 ± 2.99	14.73 ± 8.60	17.15 ± 11.20	15.58 ± 10.67	9.00 ± 2.71

*SD* standard deviation.

**Table 3:** Skinfold thickness of the examined women (mean ± SD).

Study year	Triceps skinfold	Biceps skinfold	Subscapular skinfold	Abdominal skinfold	Suprailiac skinfold	Calf skinfold
2001	21.83 ± 5.99	13.60 ± 4.99	19.71 ± 7.66	17.83 ± 5.66	24.51 ± 8.22	24.49 ± 7.16
2002	16.59 ± 5.81	10.07 ± 3.96	15.07 ± 6.34	16.22 ± 7.13	20.85 ± 7.45	18.48 ± 5.32
2003	8.16 ± 3.06	15.09 ± 6.56	15.66 ± 4.49	21.34 ± 9.09	12.06 ± 4.82	19.31 ± 5.70
2004	7.08 ± 2.80	13.36 ± 5.27	13.67 ± 4.94	20.54 ± 8.37	11.77 ± 5.57	17.21 ± 7.28
2005	8.57 ± 4.62	17.34 ± 9.24	15.85 ± 6.32	31.98 ± 10.28	12.81 ± 6.63	21.34 ± 7.08
2006	8.13 ± 2.87	16.46 ± 7.06	15.65 ± 4.58	31.69 ± 10.24	12.71 ± 6.55	17.94 ± 4.96
2007	7.56 ± 2.68	13.56 ± 4.50	15.57 ± 3.82	16.43 ± 5.77	11.75 ± 5.32	16.00 ± 4.35
2008	7.33 ± 2.51	11.78 ± 3.42	15.85 ± 3.80	13.03 ± 5.16	10.38 ± 4.21	17.85 ± 5.19
2009	7.74 ± 3.61	13.46 ± 5.50	15.64 ± 4.11	12.98 ± 5.36	10.85 ± 4.94	16.20 ± 4.64
2010	7.05 ± 2.35	12.75 ± 5.31	14.92 ± 3.96	14.23 ± 5.31	16.35 ± 6.70	16.38 ± 5.31
2011	7.85 ± 3.92	13.90 ± 5.88	15.94 ± 4.53	13.81 ± 6.63	14.06 ± 6.78	18.98 ± 5.54
2012	15.23 ± 2.99	8.11 ± 2.68	13.11 ± 4.22	13.80 ± 4.69	13.62 ± 4.60	18.26 ± 4.06
2013	15.99 ± 4.07	8.01 ± 2.76	14.00 ± 5.63	15.63 ± 4.87	15.31 ± 5.04	17.33 ± 4.22
2015	16.00 ± 4.39	7.39 ± 2.66	13.67 ± 5.94	16.27 ± 5.79	15.72 ± 6.52	17.61 ± 4.70
2016	18.38 ± 5.94	8.69 ± 3.64	16.61 ± 8.54	19.93 ± 10.23	18.61 ± 8.67	19.73 ± 6.56
2017	19.49 ± 7.12	9.31 ± 3.98	17.13 ± 8.75	21.63 ± 8.99	20.47 ± 8.69	15.59 ± 6.36
2018	15.08 ± 5.81	9.25 ± 4.74	13.80 ± 8.57	16.53 ± 6.91	16.43 ± 8.58	11.29 ± 5.35
2019	15.96 ± 5.50	9.46 ± 3.91	13.70 ± 6.17	16.58 ± 6.36	15.44 ± 7.02	11.53 ± 3.42

*SD* standard deviation.

As the data show, the examined age groups of men were characterized by lower average skinfold measurements and, with the exception of the skinfolds measured on the abdomen and on the suprailiac area, by smaller standard deviations, which confirms that the dispersion of individual measurements of somatic features in men was smaller than that in women.

### Motor skill test results

Table 4 presents the results of the fitness tests for the male students, and Table 5 presents the results for the female students.

**Table 4:** Statistical characteristics of the men in the fitness tests (mean ± SD).

Study year	Flexibility (n)	Static strength, right hand (kG)	Static strength, left hand (kG)	Trunk strength (n)	Functional strength (n)	Cardiorespiratory endurance (n)
2001	28.46 ± 5.22	51.00 ± 5.89	48.42 ± 5.71	25.75 ± 4.18	297.42 ± 238.3	8.92 ± 1.38
2002	27.00 ± 7.49	59.23 ± 6.85	55.46 ± 5.24	24.85 ± 2.85	354.23 ± 150.61	8.62 ± 1.56
2003	25.42 ± 8.00	59.50 ± 7.37	56.00 ± 8.51	25.83 ± 4.96	258.83 ± 180.73	7.50 ± 3.21
2004	21.38 ± 7.33	58.62 ± 12.22	53.62 ± 9.64	25.12 ± 3.36	205.12 ± 189.46	7.25 ± 1.67
2005	21.00 ± 7.31	47.91 ± 10.14	47.18 ± 9.12	30.36 ± 2.34	335.18 ± 157.04	8.36 ± 2.16
2006	21.41 ± 10.41	54.00 ± 9.23	50.62 ± 9.24	26.75 ± 3.33	253.12 ± 140.77	7.50 ± 1.93
2007	25.19 ± 8.21	50.70 ± 13.04	47.95 ± 10.51	28.81 ± 3.53	288.14 ± 170.18	7.78 ± 1.47
2008	24.62 ± 8.49	47.57 ± 12.31	45.86 ± 12.06	28.52 ± 3.06	279.19 ± 152.04	8.05 ± 1.91
2009	23.97 ± 7.71	57.61 ± 9.05	55.84 ± 9.20	29.65 ± 3.61	318.10 ± 109.48	8.70 ± 1.57
2010	26.26 ± 6.60	56.79 ± 9.55	56.64 ± 8.57	30.70 ± 3.39	384.39 ± 143.72	9.24 ± 1.68
2011	23.66 ± 11.42	55.86 ± 7.70	54.82 ± 9.24	28.79 ± 3.51	249.93 ± 125.51	8.50 ± 1.88
2012	25.06 ± 8.68	51.50 ± 7.26	49.03 ± 8.08	27.67 ± 3.99	341.50 ± 184.91	9.13 ± 1.78
2013	25.53 ± 7.81	55.26 ± 9.44	54.42 ± 10.43	27.68 ± 3.83	307.97 ± 164.49	8.00 ± 2.41
2015	27.73 ± 8.64	55.80 ± 9.62	53.27 ± 9.18	29.33 ± 2.89	315.87 ± 201.47	7.53 ± 1.89
2016	23.88 ± 8.36	52.73 ± 11.67	51.15 ± 11.25	28.23 ± 3.97	288.54 ± 160.14	6.88 ± 1.63
2017	26.38 ± 8.83	51.23 ± 8.88	47.46 ± 8.66	27.77 ± 3.39	232.81 ± 156.89	7.77 ± 1.45
2018	24.76 ± 9.86	56.52 ± 11.55	52.65 ± 11.20	26.35 ± 3.64	288.57 ± 154.54	7.35 ± 1.61
2019	26.56 ± 9.56	56.19 ± 10.16	53.58 ± 8.31	26.04 ± 5.59	340.42 ± 166.75	7.65 ± 2.10
2020	-	-	-	25.69 ± 3.62	-	-

*SD* standard deviation.

**Table 5:** Statistical characteristics of the women in the fitness tests (mean ± SD).

Study year	Flexibility (n)	Static strength, right hand (kG)	Static strength, left hand (kG)	Trunk strength (n)	Functional strength (n)	Cardiorespiratory endurance (n)
2001	31.60 ± 5.90	32.26 ± 4.25	29.06 ± 4.45	21.46 ± 3.43	97.43 ± 99.49	5.89 ± 1.5
2002	32.56 ± 7.04	38.15 ± 5.37	35.07 ± 4.85	22.59 ± 3.55	62.30 ± 80.10	6.22 ± 1.65
2003	30.00 ± 7.73	34.12 ± 5.42	31.56 ± 4.86	24.84 ± 2.81	149.09 ± 106.91	4.97 ± 1.45
2004	28.44 ± 6.91	35.77 ± 4.21	33.67 ± 4.75	24.05 ± 3.89	138.87 ± 116.75	5.26 ± 1.77
2005	29.36 ± 6.66	32.02 ± 5.04	30.45 ± 5.30	26.17 ± 3.55	146.32 ± 112.89	4.96 ± 1.27
2006	27.83 ± 6.39	33.56 ± 5.25	32.37 ± 5.01	24.63 ± 3.04	97.50 ± 60.54	5.23 ± 1.35
2007	27.96 ± 6.77	27.10 ± 7.03	25.39 ± 6.56	25.18 ± 2.74	128.48 ± 108.05	5.27 ± 1.54
2008	27.01 ± 5.52	28.12 ± 5.91	25.62 ± 5.90	23.05 ± 4.52	153.82 ± 124.45	5.92 ± 1.46
2009	27.27 ± 6.15	36.10 ± 9.76	35.58 ± 11.02	25.50 ± 3.55	127.32 ± 92.23	5.92 ± 1.52
2010	25.66 ± 6.16	34.37 ± 5.75	33.81 ± 5.22	25.62 ± 2.49	137.66 ± 92.30	6.30 ± 1.31
2011	30.25 ± 7.27	35.10 ± 4.82	33.83 ± 4.68	24.50 ± 4.05	114.06 ± 86.43	5.81 ± 1.33
2012	26.56 ± 5.59	33.06 ± 5.53	32.83 ± 5.73	24.08 ± 2.86	113.11 ± 109.09	6.06 ± 1.38
2013	26.01 ± 7.64	33.91 ± 5.16	33.57 ± 5.55	25.03 ± 3.54	82.90 ± 81.94	5.40 ± 1.61
2015	26.84 ± 6.12	34.28 ± 5.50	33.22 ± 5.42	24.33 ± 4.07	88.92 ± 86.96	5.34 ± 1.38
2016	29.41 ± 6.68	33.91 ± 6.10	32.66 ± 6.04	25.59 ± 3.70	116.33 ± 111.39	5.92 ± 1.36
2017	26.51 ± 6.36	31.65 ± 5.25	29.33 ± 5.05	24.92 ± 3.99	102.35 ± 123.02	5.17 ± 1.52
2018	27.56 ± 8.61	32.75 ± 6.06	31.11 ± 5.27	21.96 ± 4.11	101.87 ± 112.70	4.75 ± 1.39
2019	28.74 ± 6.38	35.61 ± 6.13	33.51 ± 6.08	23.51 ± 3.67	122.91 ± 100.65	5.07 ± 1.36
2020	-	-	-	20.33 ± 3.02	-	-

*SD* standard deviation.

The statistical data presented in the tables show that, except for flexibility, the surveyed age groups of men were characterized by higher average results of the fitness tests, and with the exception of the trunk strength, by larger standard deviations, which indicate a greater dispersion of individual results of fitness tests in men than in women. Those surveyed in 2020, especially women, were characterized by a lower average trunk strength than the student groups in earlier years.

### Data analysis

The relationships of the changes in the average annual values of the examined characteristics between women and men were assessed with Pearson’s linear correlation coefficients, and the results are presented in Table 6.

**Table 6:** Relationships of changes in average annual values of the study characteristics between females and males, as determined by Pearson’s linear correlation coefficients.

Factors	Pearson’s linear correlation coefficient	p
Age	**0.909**	**0.001**
Body height	**0.612**	**0.001**
Body mass	0.190	0.436
Triceps skinfold	**0.965**	**0.001**
Biceps skinfold	**0.884**	**0.001**
Subscapular skinfold	0.280	0.260
Abdominal skinfold	**0.934**	**0.001**
Suprailiac skinfold	**0.893**	**0.001**
Calf skinfold	**0.895**	**0.001**
Flexibility	**0.072**	**0.007**
Static strength, right hand	**0.793**	**0.001**
Static strength, left hand	**0.806**	**0.001**
Trunk strength	**0.674**	**0.002**
Functional strength	-0.141	0.576
Cardiorespiratory endurance	**0.648**	**0.004**

Statistically significant correlations are indicated in bold (p < 0.05).

With the exception of Pearson’s linear correlation coefficients calculated for the body mass, skinfold under the scapula and functional strength, all others were statistically significant, indicating that the vast majority of changes in the characteristics under observation were similar between men and women.

**Figure 1:**
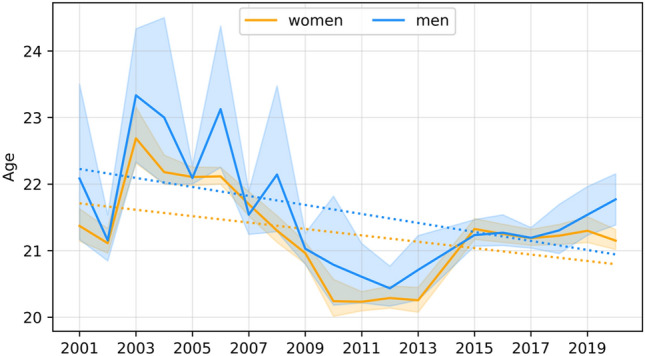
Graphical characterization of secular trends in age.

**Figure 2:**
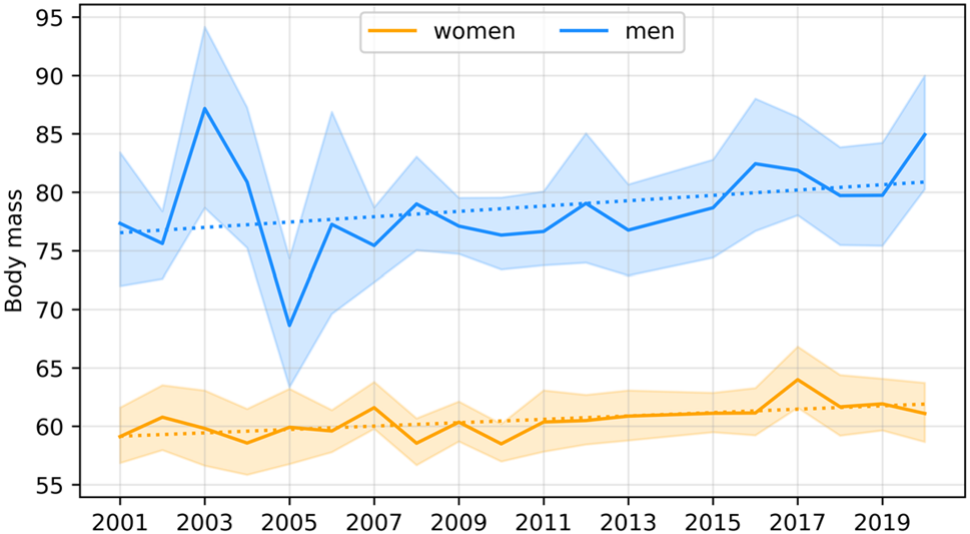
Graphical characterization of secular trends in body mass.

**Figure 3:**
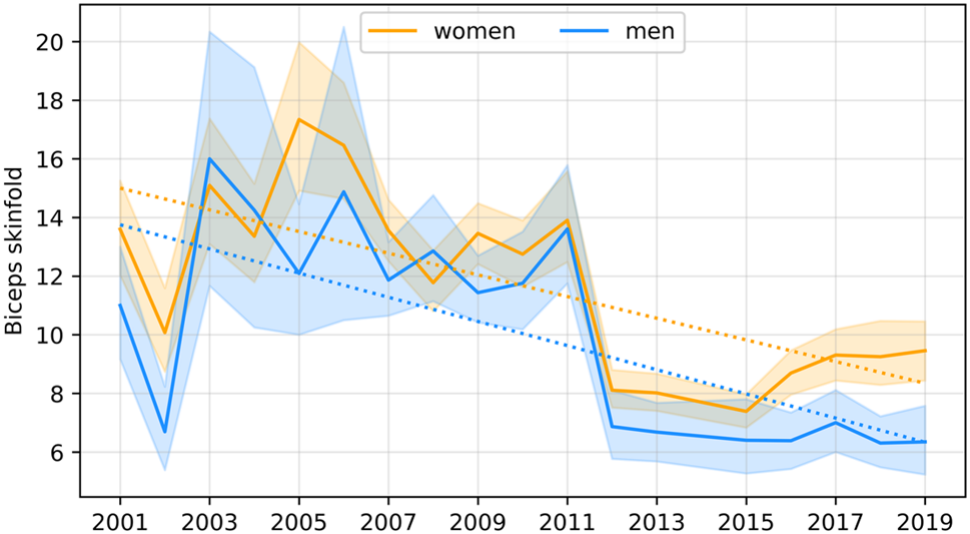
Graphical characterization of secular trends in the biceps skinfold.

**Figure 4:**
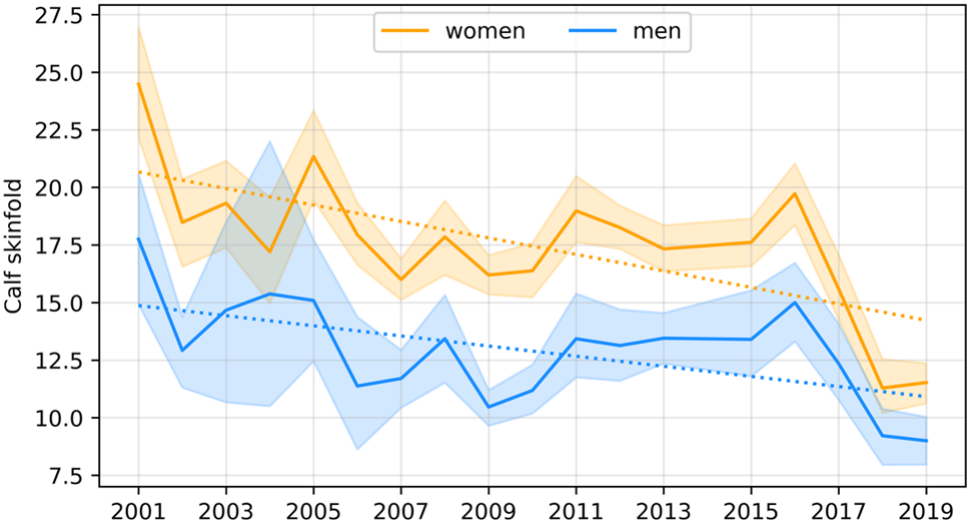
Graphical characterization of secular trends in the thickness of calf skinfolds.

**Figure 5:**
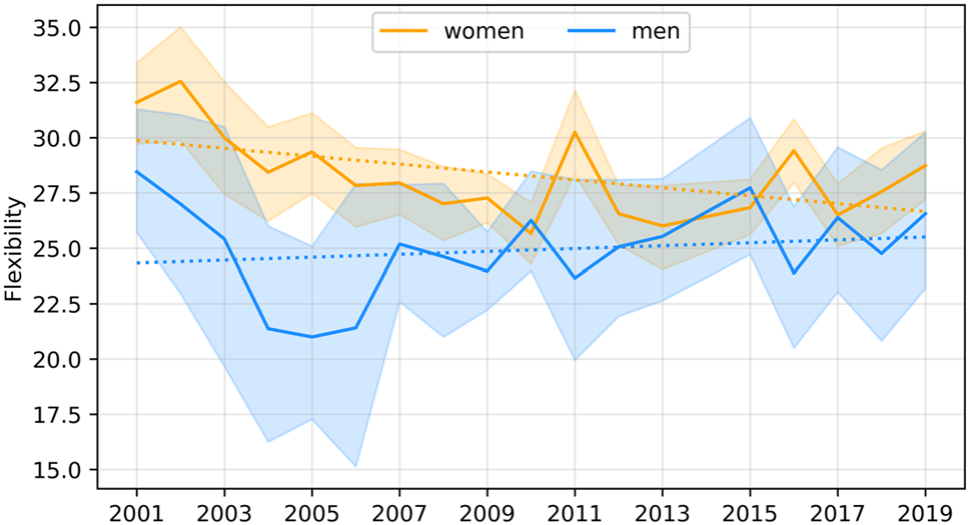
Graphical characterization of secular trends in body flexibility.

The dependence of the studied features on time was assessed using a simple linear regression model, with the coefficients for men presented in Table 7 and those for female students presented in Table 8. Significant secular trends are graphically illustrated in Figs. 1, 2, 3, 4 and 5.

**Table 7:** Coefficients of linear regression models of the form feature = b time + const for male characteristics.

Factors	time coef (b)	time p value	const	const p value	R-square
**Age**	**− 0.068**	**0.037**	**157.496**	**0.018**	**0.231**
Body height	− 0.007	0.931	− 145.658	533.952	0.001
Body mass	0.229	0.146	− 1016.516	255.258	0.12
Triceps skinfold	0.141	0.341	− 889.603	338.809	0.057
**Biceps skinfold**	**− 0.412**	**0.002**	**357.998**	**1318.973**	**0.455**
Subscapular skinfold	0.137	0.186	− 683.767	159.182	0.107
Abdominal skinfold	− 0.144	0.631	− 943.594	1556.584	0.015
Suprailiac skinfold	0.004	0.985	− 939.189	950.772	0.001
**Calf skinfold**	**− 0.220**	**0.015**	**108.850**	**799.697**	**0.314**
Flexibility	0.065	0.485	− 494.775	282.560	0.031
Static strength, right hand	− 0.014	0.933	− 606.208	770.298	0.001
Static strength, left hand	0.017	0.911	− 606.208	770.298	0.001
Trunk strength	0.031	0.671	− 337.577	268.511	0.011
Functional strenght	0.675	0.741	− 9605.655	7485.199	0.007
Cardiorespiratory endurance	− 0.038	0.200	− 36.907	206.885	0.101

Time coef—Coefficient for the time variable (b); time p value—p value for the time variable; const –value of the constant; const p value—p value for the constant; R-square—coefficient of determination.

Statistically significant models are marked with bold (p < 0.05).

**Table 8:** Coefficients of linear regression models of the form feature = b time + const for female characteristics.

Factors	time coef (b)	time p value	const	const p value	R-square
Age	− 0.048	0.077	9.716	226.179	0.173
Body height	0.051	0.289	− 130.475	261.007	0.066
**Body mass**	**0.145**	**0.003**	**− 408.887**	**− 51.842**	**0.41**
Triceps skinfold	0.322	0.144	− 1526.330	258.893	0.128
**Biceps skinfold**	**− 0.370**	**0.001**	**342.873**	**1166.861**	**0.477**
Subscapular skinfold	− 0.106	0.120	− 46.653	501.819	0.144
Abdominal skinfold	− 0.258	0.293	− 473.112	1544.738	0.069
Suprailiac skinfold	0.050	0.769	− 803.897	632.111	0.006
Calf skinfold	− 0.358	0.003	310.679	1161.976	0.445
**Flexibility**	**− 0.179**	**0.023**	**84.175**	**690.478**	**0.283**
Static strength, right hand	− 0.002	0.984	− 458.367	535.062	0.001
Static strength, left hand	0.064	0.617	− 632.452	438.144	0.016
Trunk strength	− 0.038	0.545	− 162.872	365.511	0.022
Functional strenght	− 0.655	0.553	− 3169.726	6035.545	0.022
Cardiorespiratory endurance	− 0.018	0.388	− 45.250	129.331	0.047

Time coef—Coefficient for the time variable (b); time p value—p value for the time variable; const –value of the constant; const p value—p value for the constant; R-square—coefficient of determination.

Statistically significant models are marked with bold (p < 0.05).

As the data for the age of men show, the model is significant and indicates a decrease of 0.068 years in the average age of students, along with the progressive change of the surveyed age groups. Regarding the age of women, the model is not significant and indicates that the values of the trait are maintained at similar levels.

For the body height of men and women, the models are not significant and indicate that the values of the feature are maintained at similar levels. For men’s body mass, the model is not significant, but for that of women, the model is significant and indicates a year-on-year increase of 0.145 kg in the average value of this feature^14^.

For the thickness of the skinfold at the front of the arm in men, the model is significant and shows a year-on-year decrease of 0.412 mm in the value of this feature. For women, the model is also significant and indicates a year-on-year decrease of 0.37 mm in the value of this feature.

For the remaining skinfold thicknesses of men and women, with the exception of that above the calf in men, the models are not significant and indicate that the values of these features remain at similar levels. For men’s calf skinfolds, the model is significant and shows a year-on-year decrease of 0.22 mm in this feature.

For motor tests, with the exception of women’s flexibility, the models are not significant and indicate that the values of these features remain at similar levels. For men’s body flexibility, the model is not significant, while for that of women, the model is significant and indicates a year-to-year decrease of 0.179 cm in the average value of this feature.

Multiple linear regression was also used to examine which of the somatic and sociometric characteristics adopted for the analysis significantly affected the results of the fitness tests, provided that all other indicators were the same. The most important features were selected using the sequential feature selection “forwards” method, maximizing the R-square index. Its value and the significance level of the regression models ≤ 0.001 determined the predictors, as shown in Table 9.

**Table 9:** Coefficients of significant linear regression models for the motor characteristics of the examined team.

	coef	P >|t|		[0.025	0.975]
Flexibility					
Const	51.612	0.000		44.260	58.964
Body height	− 0.144	0.000		− 0.187	− 0.101
Static strength, right hand					
Const	20.052	0.000		17.729	22.375
Body mass	0.220	0.000		0.182	0.257
Male sex	17.269	0.000		16.214	18.324
Static strength, left hand					
Const	16.863	0.000		14.520	19.206
Body mass	0.324	0.000		0.278	0.370
Subscapular skinfold	− 0.298	0.000		− 0.373	− 0.223
Male sex	13.886	0.000		12.642	15.129
Trunk strength					
Const	26.222		0.000	25.805	26.639
Suprailiac skinfold	− 0.115		0.000	− 0.139	− 0.092
Male sex	3.532		0.000	3.121	3.943
Functional strength					
Const	332.620	0.000		296.309	368.930
Body mass	− 2.805	0.000		− 3.480	− 2.131
Suprailiac skinfold	− 3.125	0.000		− 3.984	− 2.265
Male sex	231.972	0.000		214.091	249.853
Cardiorespiratory endurance					
Const	6.942	0.000		6.757	7.127
Abdominal skinfold	− 0.049	0.000		− 0.059	− 0.039
Suprailiac skinfold	− 0.035	0.000		− 0.047	− 0.024
Male sex	2.432	0.000		2.265	2.598

coef—linear regression model coefficient of the variable. P >|t|—p value of the linear regression model coefficient. [0.025–0.975]—Upper and lower bounds of the 95% confidence interval for the linear model coefficient.

For body flexibility, R-square = 0.027, p = 8e-11, and the model explained 2.7% of the variability, with a significance of 0.001 for the whole model. Increasing the body height by 1 cm reduced the flexibility score by − 0.144 cm.

For the static force of the right hand, R-squared = 0.631, p = 0.0, and the model explained 63.1% of the variability, with a significance of 0.001 for the whole model. Increasing the body mass by 1 kg increased the static force by 0.2196 kG, and male sex increased the static force by 17.269 kG.

For the static force of the left hand, R-square = 0.627, p = 0.0, and the model explained 62.7% of the variability, with a significance of 0.001 for the whole model. Increasing the body mass by 1 kg increased the static force by 0.3236 kG. Increasing the value of the scapular fold by 1 mm caused a decrease in the static force of − 0.2977 kG, and male sex increased the static force by 13.8856 kG.

For the abdominal muscle strength, R-square = 0.214, p = 3.35e-81, and the model explained 21.4% of the variability, with a significance of 0.001 for the whole model. Increasing the value of the suprailiac skinfold by 1 mm caused a decrease in the abdominal muscle strength of − 0.1154 points, and male sex increased the abdominal muscle strength by 3.5319 points.

For the functional strength, R-square = 0.424, p = 2.89e-183, and the model explained 42.4% of the variability, with a significance of 0.001 for the whole model. Increasing the body mass by 1 kg caused a decrease in the functional strength of − 2.8054 points, and an increase in the thickness of the hip fold of 1 mm reduced the functional strength by − 3.1247 points.

For the strength, R-square = 0.451, p = 5.57e-199, and the model explained 45.1% of the variability, with a significance of 0.001 for the whole model. Increasing the value of the abdominal skinfold variable by 1 mm caused a decrease in the strength of − 0.0491 points, and an increase in the thickness of the hip fold of 1 mm resulted in a decrease in the strength of − 0.0353 points. Male sex increased the strength score by 2.4319 points.

As the list shows, only male sex, body mass and the thickness of suprailiac skinfolds are frequent predictors of the examined motor characteristics that are conducive to health. However, socioeconomic determinants, i.e., the size of the place of residence, the type of school and parents’ education, as well as the inclusion of the fitness exam in the recruitment procedure for the university, did not significantly contribute to maintaining the level of the motor characteristics.

## Discussion

In the present study, a slightly different picture of the secular trend changes in the components of physical fitness supporting health in young adults was obtained compared with that based on the material collected in the analysis of the components supporting physical fitness^18^. Thus, the lack of a clear relationship between the energy factor and health and the need to separate the autotelic and heterotelic interpretations of the results of fitness tests were indirectly confirmed^20,23,26^. The results, except for body flexibility, were more favourable in the male group, which is consistent with the literature data^27,28^. This may suggest that men, who maintain better health than women, will be able to meet therapeutic standards for a longer time, which may cause numerous overloads and injuries in this profession^29,30^.

The assessment of the relationships of changes in the average annual values of the examined characteristics between women and men, with the exception of three characteristics, showed similar trends, which indicates similar reactions of the organisms of men and women to exogenous factors occurring during the course of studies. These and the previously presented research results are consistent with the observation of physical fitness components supporting motor achievements^19^. Thus, the lack of similarity in the changes in the average annual values of the observed characteristics between women and men was negatively verified, and the differences in the functional strength trends are probably related to the moderate mass gain in women, with a relative stability of this characteristic in men.

However, the main aim of this study was to determine the magnitude of changes in the examined somatic features and indicators of motor skills conducive to health over a period of two decades of observation. For the observed somatic features, secular trends of the decreased age in men and increased body mass in women, as well as the stability of the body height in both sexes, were found in the student groups^18^. In terms of the body height and mass of female students, the results obtained do not clearly correspond to those of secular trends in other student groups, especially those studied in earlier decades. Previously, secular trends of increasing body height and mass, accompanied by a weaker body structure, were found^15,31,32^. Some of the studies showed a secular trend of an increasing BMI among Polish students, which contradicts the trends for this indicator in the currently analysed groups of female students in the 2011–2020 decade^18,32^. Among the currently examined characteristics, there were also trends towards a decrease in the thickness of the skinfold over the biceps and of that measured over the calf among men, which may indicate a greater activity involving the limbs, especially the upper limbs, than that involving the other parts of the body. It is also possible that there are tendencies for central distribution of adipose tissue and its reduction in the distal parts, which is beneficial in the case of the upper limbs^33,34^. There is scientific evidence of a secular trend of an increasing thickness of skinfolds and their stabilization on the upper torso, as well as increasing obesity among academic youth^13,31^. The reduction in the thickness of skinfolds on the lower leg among the currently studied students should be considered an unfavourable change, especially in light of the significant increase in their waist circumference during the pandemic^18^. This may increase the future risk of cardiovascular disease, especially when combined with the central fat allocation. It can also be a component of insulin resistance, leading to diabetes, hyperlipidaemia and hypertension^35^. However, this statement is of relative value because it is based on the observations of the skinfold thickness and waist circumference and not on detailed measurements of body components.

An increase in women’s body mass, with a significant reduction in the thickness of skinfolds at the front of the arm and a trace of this tendency in most other folds, without body height changes, may indicate the muscle mass growth in female students or a general tendency for a slimmer figure and a reduced percentage of obesity in many communities, especially in young ones^36–40^. We consider the above changes to be beneficial, which was probably reflected in the stability of the vast majority of the examined indicators of motor skills. This observation contradicts scientific evidence showing an increase in the thickness of skinfolds or an increase in obesity presented in other studies^15,49^.

It is disturbing, however, that the level of body flexibility in women significantly deteriorated, although women are more predisposed to its manifestation than men are. This may indicate a lack of care by women for this feature, which, without targeted exercise, deteriorates with age. It is therefore possible that in subsequent professional work, women will be more exposed to overloads and injuries of the lower part of the spine, which are becoming common phenomena among physiotherapists^28^. We base this assumption both on general knowledge concerning the interpretation of the results of flexibility tests and on reports on the occurrence of pain in the lumbosacral spine among physiotherapy students^41^. It is also possible that the deterioration of women’s flexibility, in addition to the effects of the pandemic, affected the unfavourable trunk strength results obtained in 2020. However, this assumption requires continued research taking into account the effects of the pandemic and the history of SARS-CoV-2 infection.

However, the presented negative secular trends were smaller than expected, especially in the context of numerous reports of deteriorating physical fitness and health status in many societies^6,7,14,32^. Thus, the hypothesis about the deterioration of the components of physical fitness that support the health of young adults over time was mostly negatively verified. Nevertheless, it should still be taken into account that apart from statistically significant secular trend changes over time, there also were trends of deterioration in the levels of static strength of the right hand and endurance in both sexes, as well as in the strength of the abdominal muscles and functional strength in women, which may have intensified over time. We base this supposition on the results of many studies that showed favourable changes in physical development and simultaneous deterioration of physical fitness in young generations^42–45^. Therefore, special attention should be given to the improvement of the examined components of physical fitness, especially the grip strength, which is an important indicator of energy abilities and can be used in predicting health and well-being, especially in older people^46^. In this study, the results obtained among the studied groups of students did not correspond to the scientific literature data^47,48^. With clearly different levels of static strength between the examined and compared groups, the information about the significantly lower strength of the dominant hand was not confirmed either. The vast majority of the people surveyed by us were right-handed, and in all age groups of both men and women, the grip strength of the right hand was slightly greater than that of the left hand. Therefore, we believe that differences in the procedures of the performed tests and the significantly larger number of groups examined in this study than in the other studies contributed to the observed difference.

It was not possible to unambiguously determine the predictors of the observed characteristics. Only male sex, body mass and the thickness of suprailiac skinfolds were found to be predictors of several examined motor characteristics, with no influence of sociometric determinants, including the inclusion of the fitness exam in the university recruitment procedure, although two of the three fitness tests, handgrip and endurance shuttle run, were within the scope of the present study. Thus, our results are somewhat surprising and allow us to claim that the too low criteria of the fitness exam were not sufficiently selective for physically fit people, while intellectual selection for studies was the decisive factor and was thus somewhat discriminatory against candidates from lower social class levels. Hence, there was little social diversity among the studied students, and therefore, there were no sociometric features among the predictors of physical fitness tests supporting health.

The presented research results represent only a fragment of the secular trends in the physical fitness of the surveyed physiotherapy students. In addition to the currently studied components, physical fitness includes other components supporting motor achievements, certain body structure regularities, and daily physical activity and skills^20^. In the profession of physiotherapists, these components are of particular value because they allow the use of various types of physical activity in the broadly understood therapeutic process. Thus, the continuation of research allowing for recognition of the area of physical activity and motor skills as well as students’ readiness to later use them in their professional work seems fully justified. Issues worth considering in subsequent studies also include the further effects of the pandemic and social changes caused by warfare in Europe.

During the implementation of the present research, certain limitations were encountered, which make the results only relatively relevant. These include the fact that the research covered students from only one medical university and those studying at different levels of education and years of study, as well as the impossibility of conducting research during the lockdown period. Covering first-year students in the study may have obscured the actual results because of the need for young people to adapt to a new reality, which often causes heavy burdens and negative dietary changes, as well as a reduction in recreational and sports activities^49,50^. The small number of men in the groups could also have influenced the observed secular trend of the reducing age of the students. However, we believe that the advantage of this work is the relatively long duration of research carried out with a recognized physical fitness test by one research team, as well as the ability to obtain complete results from a large percentage of students under observation. It was also important to undertake and continue a study, starting from the first recruitment of physiotherapy students at the Polish Medical Academy. Therefore, we believe that the obtained results are relatively reliable and may be helpful for updating physical education programs in physiotherapy studies and for recruiting future candidates. The results may also provide valuable information material for people intending to study physiotherapy.

## Conclusions

The results of many years of observation of trends in secular components of physical fitness supporting the health of people studying physiotherapy at the Medical University of Bydgoszcz showed a trace of a negative tendency in changes in all examined motor characteristics. The exceptions were several identified somatic features and the deterioration of flexibility in women. Therefore, attention to the appropriate level of the observed features seems to be justified, and the results of the present research may be helpful in updating physical education programs in physiotherapy studies or in selection of candidates for such studies based on their motor skills. It also seems advisable to continue the research topic, extending it to the area of motor skills, which are useful both in undertaking targeted physical activity and in creating kinesiotherapy exercises conducted with patients.

## Data Availability

The datasets used and/or analysed during the current study will be made available from the corresponding author upon reasonable request, with the consent of the Bioethics Committee of the Collegium Medicum in Bydgoszcz.
